# The uniqueness of human vulnerability to brain aging in great ape evolution

**DOI:** 10.1126/sciadv.ado2733

**Published:** 2024-08-28

**Authors:** Sam Vickery, Kaustubh R. Patil, Robert Dahnke, William D. Hopkins, Chet C. Sherwood, Svenja Caspers, Simon B. Eickhoff, Felix Hoffstaedter

**Affiliations:** ^1^ Institute of Systems Neuroscience, Medical Faculty and University Hospital Düsseldorf, Heinrich-Heine-University, Düsseldorf, Germany.; ^2^ Institute of Neuroscience and Medicine (INM-7), Research Center Jülich, Jülich, Germany.; ^3^ Division of Physiotherapy, Department of Applied Health Sciences, Hochschule für Gesundheit (University of Applied Sciences), Bochum, Germany.; ^4^ Structural Brain Mapping Group, Department of Neurology, Jena University Hospital, Jena, Germany.; ^5^ Structural Brain Mapping Group, Department of Psychiatry and Psychotherapy, Jena University Hospital, Jena, Germany.; ^6^ Center of Functionally Integrative Neuroscience, Department of Clinical Medicine, Aarhus University, Aarhus, Denmark.; ^7^ Department of Comparative Medicine, Michale E. Keeling Center for Comparative Medicine and Research, The University of Texas MD Anderson Cancer Center, Bastrop, TX, USA.; ^8^ Department of Anthropology and Center for the Advanced Study of Human Paleobiology, The George Washington University, Washington, DC, USA.; ^9^ Institute of Neuroscience and Medicine (INM-1), Research Center Jülich, Jülich, Germany.; ^10^ Institute for Anatomy I, Medical Faculty and University Hospital Düsseldorf, Heinrich-Heine-University, Düsseldorf, Germany.

## Abstract

Aging is associated with progressive gray matter loss in the brain. This spatially specific, morphological change over the life span in humans is also found in chimpanzees, and the comparison between these great ape species provides a unique evolutionary perspective on human brain aging. Here, we present a data-driven, comparative framework to explore the relationship between gray matter atrophy with age and recent cerebral expansion in the phylogeny of chimpanzees and humans. In humans, we show a positive relationship between cerebral aging and cortical expansion, whereas no such relationship was found in chimpanzees. This human-specific association between strong aging effects and large relative cortical expansion is particularly present in higher-order cognitive regions of the ventral prefrontal cortex and supports the “last-in-first-out” hypothesis for brain maturation in recent evolutionary development of human faculties.

## Teaser

Humans and chimpanzees show brain aging, while its spatial distribution relates to evolutionary expansion only in humans.

## INTRODUCTION

With age, pronounced alterations occur in morphology and organization of the human brain with a distinct spatial pattern resulting in part from cellular atrophy in later life (*1*, *2*). This aging process may be further accelerated by age-mediated disorders such as Alzheimer’s disease, Parkinson’s disease, and other neurodegenerative conditions (*3*). Furthering our understanding about specific neurobiological influences on spatial patterns of brain aging may provide insight into the brain changes in healthy aging and possible diagnostic markers for clinical outcomes. Historically, comparative neuroscience has been an effective catalyst for important discoveries regarding principles of anatomy and functional specializations of the human brain (*4*). With open and collaborative endeavors such as the National Chimpanzee Brain Resource (NCBR) and the PRIMatE Date Exchange (*5*), along with improved methodologies and imaging technology, large-scale comparative neuroanatomy has become able to answer new translational questions (*6*).

Morphological gray matter (GM) changes during aging have recently been shown to be present in one of humans’ closest extant primate relatives, chimpanzees (*Pan troglodytes*) (*7*, *8*), where age-related changes are similar but at a lower magnitude compared to humans (*8*). For example, age-related volumetric reduction of overall hippocampus and frontal cortex size is not evident in chimpanzees but occurs in humans. Cognitive decline is also present in chimpanzees but appears not as prominent as in humans (*9*). It has been proposed that these differences in neurobiology of aging might be related to the extended life span in humans (*10*). In this context, understanding GM alterations during brain aging in great ape evolution (e.g., which includes humans and chimpanzees, as well as bonobos, gorillas, and orangutans) may aid in understanding the spatial distribution of morphological changes due to healthy aging and disease.

The comparison of neuroanatomy and brain functions across primate species is commonly informed by analyzing homologous brain regions (*6*, *11*–*13*). Classically, these regional homologies are defined by manually delineating brain partitions, based on macroanatomy, gene expression, connectivity, and/or cytoarchitectonic features. This approach rests on the assumption that similar anatomical features result in a common functional organization across species and thereby enable an informative and meaningful comparison between them. However, such homologies can be contentious, sometimes influenced, for example, by methodological biases (*14*), like until recently the delineation of the prefrontal cortex (PFC) in primates (*11*). Through combination of sulcal pattern analysis with resting-state functional magnetic resonance imaging (MRI) and cytoarchitectonic analysis, there is good evidence for specific sucli delineating the border between prefrontal and premotor cortices in four primate species (*15*). The homologous-centric approach has proven to be effective and informative. Using a data-driven approach can supplement these techniques while still capturing important cross-species differences and incorporating species-specific features in a data-centric manner (*16*).

Chimpanzees offer an ideal referential model to investigate evolutionary changes within the human lineage as they share a last common ancestor with humans approximately 6 to 8 million years ago (*17*). Accordingly, chimpanzees and humans have substantial genomic similarities (*18*) as well as neuroanatomical features in common (*19*–*21*). Furthermore, new evidence suggests that menopause occurs at a similar age in humans and chimpanzees, with demographic and hormonal data indicating that reproductive cessation in both species is caused by a common physiological factor (*22*), although human life spans have the potential to extend substantially longer past the age of menopause than in chimpanzees. Consequently, chimpanzees represent a unique possibility to infer distinctive evolutionary adaptations of the human brain by analyzing commonalities and recent divergences. Previous studies have shown that multimodal association cortices in humans are disproportionately larger than in nonhuman primates (*11*, *23*, *24*). The higher expansion of certain brain areas through human evolution likely relates to human-specific cognitive functions. Specifically, the greatly expanded human PFC (*11*) can be associated with self-control and executive functioning (*25*) and the larger precuneus (*23*) with visuospatial processing (*26*). Furthermore, the vulnerability of frontal cortical areas to aging processes is hypothesized to be related to their late maturation (*27*). This refers to the “last-in-first-out” hypothesis, and interesting similarities have been shown between cortical development and cross-species expansion (*28*).

In this study, we directly compare age-mediated GM changes in chimpanzees and humans, which represent two species in the Hominidae family (i.e., great apes) and explore their relationship with cross-species cerebral expansion. For interspecies comparison, we developed a multivariate data-driven comparative framework that applies voxel-wise clustering based on GM variability within each species independently. The optimal low-dimensional representation of brain morphology for each species is then compared in a cross-species investigation of aging and brain expansion. Comparative data for calculating cross-species expansion was provided via select phylogenetic relatives. Accordingly, humans were compared to chimpanzees, while chimpanzees were compared to olive baboons (*Papio anubis*) and rhesus macaques (*Macaca mulatta*), two commonly researched cercopithecoid monkey species. Thus, we test whether the relationship between aging and cerebral expansion is unique to humans or instead might be a feature shared between humans and chimpanzees possibly originating at the divergence of the great ape lineage from other primates.

In summary, we present a data-driven cross-species comparison of structural brain organization and demonstrate its utility by analyzing the relationship between cerebral aging and cross-species expansion in humans and chimpanzees. Our data-driven approach uses both species-specific information and cross-species similarity to create an anatomically interpretable low-dimensional brain parcellation. We show that the resulting parcellation aligns with known macroanatomical structures in both humans and chimpanzees. Applying this comparative framework, we jointly analyze spatial patterns in brain aging and cerebral expansion of the two great ape species. Last, we present evidence for a relationship between local age-mediated GM changes and recent cortical expansion in humans that is not present in chimpanzees.

## RESULTS

**Fig. 1.**
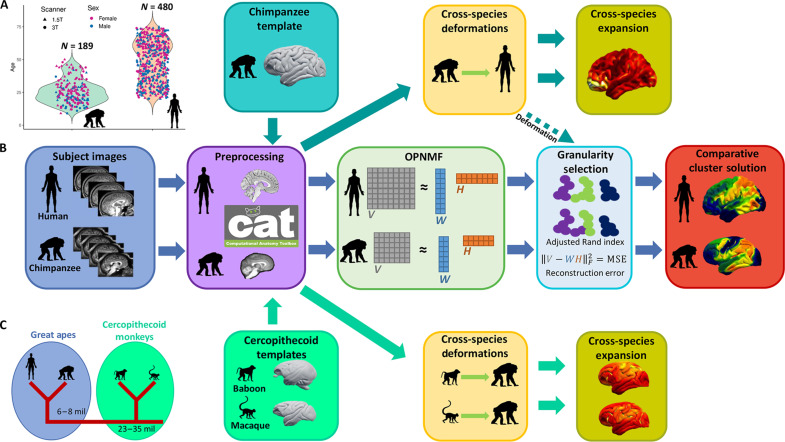
Sample, workflow, and phylogeny. (**A**) Age, sex, and scanner field strength distribution of the chimpanzee (*N* = 189) and human (*N* = 480) samples. (**B**) Workflow outlining our comparative approach by using OPNMF and creating cross-species expansion maps. (**C**) Diagram showing the phylogenetic relationship of humans to the other three primate species investigated in this study.

Our cross-species comparative approach was based on structural MRI scans from 189 chimpanzees and 480 human brains (Fig. 1B). Orthogonal projective non-negative matrix factorization (OPNMF) (*29*, *30*) was applied to normalized GM maps within each species independently. The orthogonality and non-negativity constraints of OPNMF results in a spatially continuous, part-based representation of the input data based on regional covariance of brain structure within each species (*31*). OPNMF has been extensively used with human neuroimaging data yielding anatomically meaningful correspondence of clustering solutions (*29*, *32*–*35*).

The comparative framework using OPNMF as well as the creation of the cross-species expansion maps is outlined in Fig. 1A. The approach begins with separately segmenting and normalizing the individual chimpanzee and human images using species-specific templates in almost identical Computational Anatomy Toolbox (CAT12) pipelines (*8*, *36*). The processed GM maps for each species are parcellated independently using OPNMF over a range of granularities (*2*–*40*) and bootstrapped over the whole sample with replacement to ensure stability of the solutions. Mean reconstruction error (MRE) of each OPNMF solution over bootstraps is used to select a range of clustering solutions with optimal numbers of parcels for cross-species comparison. For direct cross-species comparison, the JunaChimp average chimpanzee T1-weighted (T1w) template (*8*) is submitted to the human preprocessing pipeline to create a representative chimpanzee to human deformation map. The JunaChimp (*8*) to human (*37*) deformation map is used to nonlinearly register the chimpanzee OPNMF solutions to the human template space for the analysis of parcel similarity using the adjusted rand index (ARI). ARI is a measurement of similarity between two clustering solutions that is corrected for chance similarity. A value of 1 represents the same clustering, while 0 indicates that there is chance level agreement in the clustering, while <0 indicates similarity of less than chance. These cross-species parcel similarity of multivariate GM morphology are used for the selection of optimal parcellation granularity, together with species-specific OPNMF MRE. To create cross-species expansion maps for chimpanzees, average population templates from olive baboon (*38*) and rhesus macaque (*39*–*41*) were processed with the chimpanzee pipeline (*8*). Therefore, the cross-species volumetric expansion maps are derived from population templates of chimpanzees and the two cercopithecoid monkeys that each provides representative brain morphology with high tissue contrast for accurate cross-species registration and deformation.

### Comparative brain parcellation

**Fig. 2.**
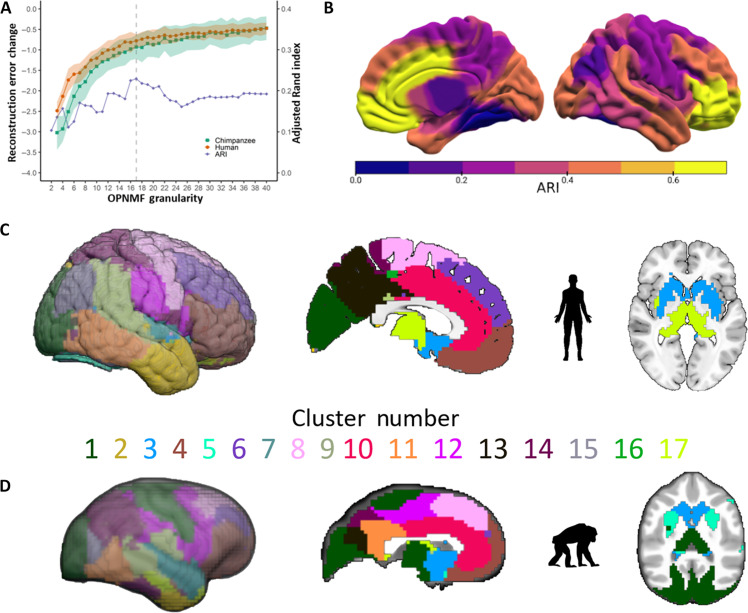
The 17-cluster OPNMF solution for cross-species comparison. (**A**) OPNMF granularity selection using ARI to assess cross-species similarity and relevant change in reconstruction error over a granularity range of 2 to 40 clusters and bootstrapped (*k* = 100) to ensure stability. The 1 SD from the change in MRE over 100 bootstraps is represented as a shadow; the gray dashed line represents the selected number of 17 clusters. (**B**) Cross-species single parcel ARI in human template space. (**C**) Human selected 17-cluster OPNMF solution with macroanatomical labels: 1, occipital lobe; 2, temporal pole; 3, putamen, caudate nucleus, amygdala, and hippocampus; 4, prefrontal and orbito-frontal cortex; 5, lingual and fusiform gyrus; 6, superior and middle frontal gyrus; 7, insula; 8, precentral gyrus and premotor area; 9, temporal parietal junction; 10, anterior and middle cingulate cortex; 11, posterior middle and inferior temporal gyri; 12, supramarginal gyrus, inferior postcentral gyrus, and inferior precentral sulcus; 13, precuneus; 14, superior parietal lobe; 15, angular and fusiform gyrus; 16, superior parietal sulcus and parahippocampal cortex; 17, thalamus. (**D**) Selected 17-cluster OPNMF solution for chimpanzees with macroanatomical labels: 1, occipital lobe, primary motor cortex, and thalamus; 2, temporal pole; 3, caudate nucleus; 4, prefrontal and orbito-frontal cortex; 5, putamen; 6, middle frontal gyrus; 7, superior temporal gyrus and anterior insula; 8, posterior superior frontal gyrus; 9, temporal parietal junction and supramarginal gyrus; 10, anterior and middle cingulate cortex; 11, posterior cingulate, precuneus, and peristriate cortex; 12, supplementary and premotor areas; 13, cuneus and medial occipital-parietal sulcus; 14, superior and inferior parietal lobe and inferior temporal gyrus; 15, lateral parietal-occipital sulcus; 16, superior parietal sulcus and posterior insula; 17, amygdala and hippocampus.

OPNMF clusters the volumetric GM maps and yields parcels which contain voxels that covary with one another across the sample. This unsupervised clustering technique behaves similar to others like independent component analysis and requires an a priori decision on the number of clusters to represent the original data (*29*). The decision for the most appropriate OPNMF solution was determined via assessing cross-species spatial similarity and the development of OPNMF reconstruction accuracy at different granularities (Fig. 2A). Chimpanzee parcellations were transformed to the human template space using the chimpanzee to human deformation map for assessing cross-species parcel similarity using ARI. Quality assurance of the chimpanzee to human deformation map was conducted by visually inspecting the overall alignment of the Davi130 chimpanzee macroanatomical labels (*8*) to known human macroanatomical landmarks by agreement of two authors (S.V. and R.D.) (fig. S1). The OPNMF solution with highest ARI for within species parcellations represents common cross-species organizational patterns of GM covariance (*31*). The MRE indicates how accurately the input data (GM maps) can be represented by the OPNMF factorization. By increasing the number of clusters, more variance in GM input data is modeled and MRE naturally decreases, while this association is nonlinear and sample specific (Fig. 2A). A plateau of the MRE decrease with increasing OPNMF granularity only marginally improves the solution’s representation of GM data. Consequently, the beginning of a plateau hints at a good tradeoff between the solutions reconstruction accuracy and the complexity of the cluster solution. Last, to ensure the robustness of the MRE development curve, 100 bootstraps were computed for each OPNMF granularity in both species.

The highest spatial similarity of parcellations between species was found for the 17-cluster solution with a mean ARI of 0.23 (Fig. 2A) including several parcels with ARI > 0.4 (Fig. 2B). The MRE development curve did not show a clear indication of a plateau for both species, yet a gradual plateau is present in the range of 15 to 21 clusters in chimpanzees and 14 to 20 in humans. Therefore, the 17-cluster solution met our criteria for both humans (Fig. 2C) and chimpanzees (Fig. 2D) for a data-driven cross-species comparative investigation.

The 17-cluster OPNMF solutions in both humans (Fig. 2C) and chimpanzees (Fig. 2D) represents a data-driven parcellation of both species’ cerebral GM. OPNMF has been successfully implemented in human brain clustering (*29*, *32*, *34*), although has yet been presented in chimpanzees. We show that the chimpanzee change in MRE follows a similar trajectory as in humans (Fig. 2A). Furthermore, the chimpanzee clusters are predominantly symmetrical across hemispheres, align with known chimpanzee macroanatomical structures, and show high spatial connectedness and smoothness. These quantitative and qualitative measures ensure that OPNMF can be effectively used to establish data-driven, anatomical valid clustering in chimpanzee brain. The OPNMF clusters closely align with known macroanatomical regions in both species like the orbito-frontal cortex, middle frontal gyrus, anterior and middle cingulate cortex, and the temporal pole (Fig. 2, C and D). In contrast, for humans, separate parcels delineated the insula, superior parietal lobule, precuneus, occipital lobe, and thalamus (Fig. 2C), while in chimpanzees, the premotor cortex, hippocampus, putamen, and caudate nucleus were differentiated (Fig. 2D). Overall, orbito-frontal and the cingulate cortices showed the highest cross-species similarity with an ARI of 0.66 and 0.64, respectively (Fig. 2B). A marked difference between species can be seen in the parcellation of sensory-motor cortices. In chimpanzees, two parcels represented major sensory-motor structures, one for the occipital lobe, pre- and postcentral gyrus, and thalamus and another for the premotor cortex. In humans, separate parcels represented thalamus and occipital lobe as well as motor and premotor cortices. Specifically, in humans, multimodal parietal regions like the precuneus, superior parietal lobule, angular gyrus, and temporal-parietal junction were parcellated into different clusters, while in chimpanzees, the basal ganglia are more differentiated.

### Brain aging

**Fig. 3.**
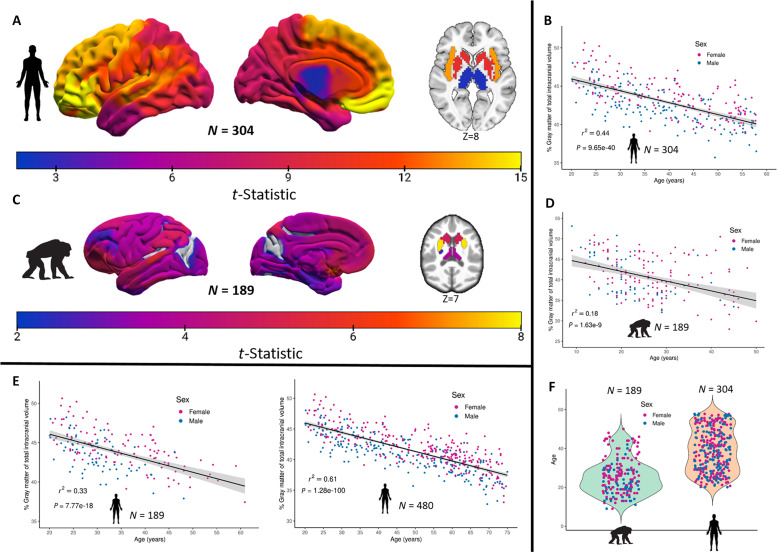
Age-related GM decline. (**A**) Maximum chimpanzee-matched age human sample (*n* = 304) significant (FWE *P* ≤ 0.05) age-mediated GM changes presented as absolute *t*-statistic from cluster-wise regression model. (**B**) Scatterplot representing percentage GM of TIV against age in maximum age-matched human sample. (**C**) Chimpanzee significant (FWE *P* ≤ 0.05) age-mediated GM changes presented as absolute *t*-statistic from cluster-wise regression model. (**D**) Chimpanzee scatterplot showing percentage GM of TIV against age. (**E**) Human 1:1 matched sample based on age, sex, and scanner field strength (*n* = 189) percentage GM of TIV age regression (left) and violin plots showing age and sex distributions in 1:1 chimpanzee human-matched samples (right). (**F**) Violin plots presenting age and sex distribution of chimpanzee and maximum age-matched human samples.

Age-mediated GM decline in chimpanzees and humans was assessed for the OPNMF 17-cluster solution. The average GM volume of each parcel was used as the dependent variable in a multiple linear regression model with age, sex, total intracranial volume (TIV), and scanner field strength as independent variables. To improve comparability, the human sample age range was matched to the chimpanzees by accounting for the interspecies differences in brain aging. The comparative aging difference of human years approximated to 1.15 years in chimpanzees was used based on a comprehensive study using a combination of anatomic, genetic, and behavioral data (*42*). Accordingly, as the oldest chimpanzees were 50 years old, humans over 58 years old were removed to include 304 subjects (150 females; mean age = 39.0 ± 11.0) to represent a maximum age-matched human sample for comparative age-mediated GM atrophy. Of note, this represents a middle-aged human sample, including minimal morphological changes due to age-related neurodegenerative or preclinical conditions such as mild cognitive impairment. Considering the early stages of brain aging in humans, this comparative sample presented significant parcel-wise (Fig. 3A) and total GM percentage of TIV (Fig. 3B; *r*^2^ = 0.44; *P* = 9.65 × 10^−40^) age-mediated GM atrophy. Chimpanzees also showed significant total GM age-related atrophy (Fig. 3D; *r*^2^ = 0.18; *P* = 1.63 × 10^−9^) and displayed significant age-mediated GM decline in all but three parcels, which represent the peristriate cortex, posterior insula, cuneus, and superior parietal sulcus (Fig. 3C). Significant OPNMF parcels are presented following correction for multiple comparisons across parcels at *P* ≤ 0.05 (*43*). Humans showed age-related GM decline across all parcels, largest in frontal and prefrontal cortices (Fig. 3A). Both species showed relatively low age-related changes in occipital and motor areas. The largest agerelated GM decline in chimpanzees was found in the striatum, in particular the caudate nucleus. Furthermore, we reproduced the significant GM atrophy finding in the maximum age-matched sample in a 1:1 matched sample based on age, sex, and scanner (Fig. 3E; *n* = 189; *r*^2^ = 0.33; *P* = 7.77 × 10^−18^) and the whole IXI (Information eXtraction from Images) sample (Fig. 3E; *n* = 480; *r*^2^ = 0.61; *P* = 1.28 × 10^−100^). The 1:1 matched human sample demonstrates the stability of the age-related GM changes in humans when considering the lower sample size and sex distribution of the chimpanzee sample. In addition, this smaller subsample presents comparable age-mediated GM atrophy as the whole IXI sample (Fig. 3E). Of note, comparable spatial distribution of age-related GM decline was found when using the same macroanatomical Davi130 parcellation (*8*) with 7.6-fold higher granularity in both chimpanzees and humans (fig. S2).

### Cross-species brain expansion

Using the 17-cluster solution, we compared cross-species brain expansion based on population representative T1w templates from humans (*37*), chimpanzees (*8*), olive baboons (*38*), and rhesus macaques (*39*–*41*). To provide estimations for cerebral expansion, we calculated cross-species nonlinear coregistration of the brain from chimpanzee to human, baboon to chimpanzee, and macaque to chimpanzee. We present the estimated cross-species expansion maps as relative expansion, where values of one represent a local volumetric expansion comparable to the overall difference in brain size between species. Therefore, values greater than one represent local brain areas that have expanded more than the overall difference in brain size between species, and values less than one show lower expansion than brain size difference.

**Fig. 4.**
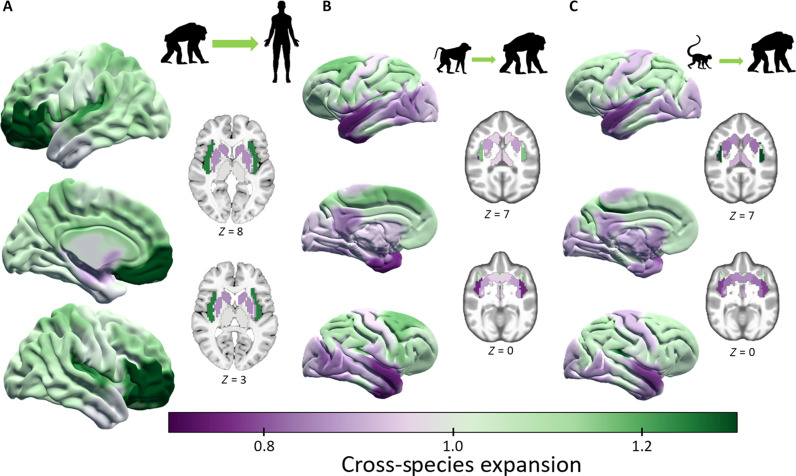
Species-specific OPNMF 17-cluster of cerebral expansion. Average relative cross-species template expansion for each OPNMF 17-cluster is shown for (**A**) human expansion from chimpanzee, (**B**) chimpanzee from baboon, and (**C**) chimpanzee from macaque expansion.

The largest human expansion was found in the orbito-frontal cortex, which additionally showed the greatest age-mediated GM decline (Fig. 3A) and also the highest cross-species parcel similarity (Fig. 2B). Further large cortical expansion was found in other multimodal association areas such as the middle and medial frontal cortex, superior parietal, precuneus, insula, and cingulate cortex. Low expansion was located in the temporal pole as well as occipital, motor, and subcortical areas (Fig. 4A). These latter regions also contained lower expansion in the baboon (Fig. 4B) and macaque (Fig. 4C) to chimpanzee expansion maps, although the cercopithecoid monkeys to chimpanzee presented much lower expansion as compared to the chimpanzee to human map. In addition, the precuneus showed high expansion in the human (Fig. 4A) and relatively low in the chimpanzee from both cercopithecoid monkeys. The general pattern of expansion in both cercopithecoid monkeys to chimpanzee is similar, with large expansions of frontal, parietal, and cingulate cortices. In baboon to chimpanzee, the largest expansion occurred in the superior frontal gyrus/premotor area (Fig. 4B), while in macaque to chimpanzee, the superior parietal sulcus and posterior insula (Fig. 4C) featured the largest expansion. Furthermore, macaque to chimpanzee showed comparably more expansion in the occipital-parietal junction and lower expansion in the motor/premotor area, occipital cortex, and basal ganglia compared with baboon to chimpanzee expansion. To summarize, chimpanzee to human as well as cercopithecoid monkeys to chimpanzee expansion maps show relatively high expansion in frontal and parietal cortical regions. The human features the greatest expansion in prefrontal areas, while in chimpanzees, the largest expansion was seen in premotor/frontal and lateral parietal regions.

### Brain aging and cross-species expansion

**Fig. 5.**
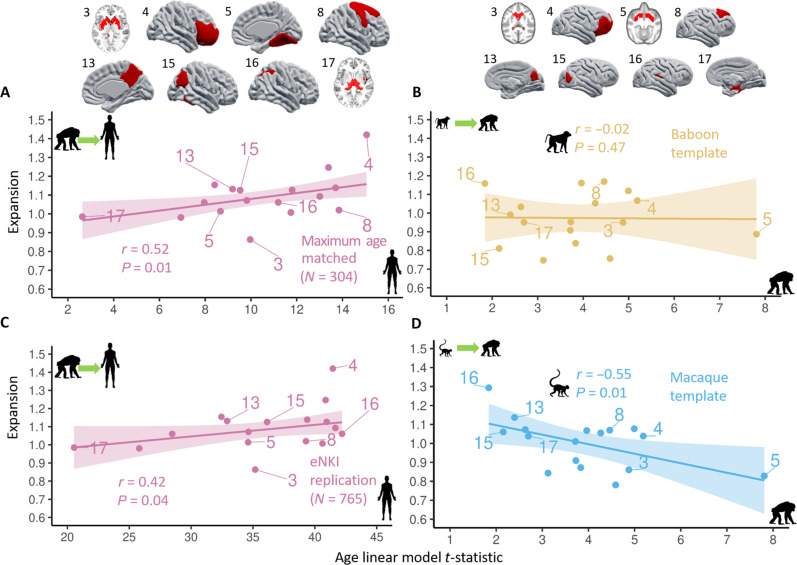
GM aging and cerebral expansion. Each dot represents an OPNMF brain parcel, and a selection of parcels for human and chimpanzee is shown above the scatterplots of (A) and (B), respectively. (**A**) Chimpanzee to human expansion and human age-related GM decline in maximum age-matched sample. (**B**) Baboon to chimpanzee expansion and (**D**) baboon to chimpanzee expansion correlated with chimpanzee age-related GM decline. (**C**) Chimpanzee to human expansion and human age-related changes relationship presented in eNKI whole life-span external replication dataset. Significance (*P*) of Person’s correlation (*r*) for cross-species expansion and age-related GM decline relationship is determined by permutation testing (*k* = 100,000).

We investigated the relationship between cross-species expansion and age-related GM changes in chimpanzees and humans. For humans, brain aging was compared with cortical expansion from chimpanzee to human, while for chimpanzees, aging was compared with expansion relative to both baboon and macaque. A strong positive correlation was found between cerebral expansion and age-mediated GM decline in humans (Fig. 5A), following permutation testing at *P* ≤ 0.05 (*r* = 0.52; *P* = 0.01). This relationship is particularly evident in the orbito-frontal cortex and insula, with considerable expansion and age-related GM decline, while low decline and expansion was found in the basal ganglia, occipital lobe, temporal pole, and medial temporal lobe. This general association was replicated in another large dataset, the eNKI (enhanced Nathan Kline Institute; *n* = 765; *r* = 0.42; *P* = 0.04) (*44*) sample using the same OPNMF parcellation to extract the age-mediated GM decline (Fig. 5C). In addition, we replicated the significant relationship between cerebral expansion and age-related GM decline in humans applying the much finer Davi130 chimpanzee parcellation (*8*) (*r* = −0.38; *P* = 1 × 10^−4^; fig. S3A). Furthermore, using the parcel-wise age-related GM changes from the 1:1 human-matched sample (*n* = 189), the relationship between aging and expansion was found to be slightly higher (*r* = 0.55; *P* = 0.01; fig. S4) than the maximum age-matched (*n* = 304) human sample. Therefore, we have demonstrated the stability of the aging-expansion relationship in humans in an external large human dataset (eNKI), with increased brain parcellation granularity (Davi130), and in a 1:1 cross-species matched sample.

In chimpanzees, no significant relationship was observed between aging and cerebral expansion from baboon (Fig. 5B; *r* = −0.02; *P* = 0.47), but a negative correlation was found for expansion from macaque (Fig. 5D; *r* = −0.55; *P* = 0.01). Although the macaque and baboon to chimpanzee expansion maps show a similar pattern in spatial distribution, there is an apparent trend of regions showing greater macaque to chimpanzee expansion in regions with minimal age-related GM decline. This relationship is strongly driven by the greater macaque to chimpanzee expansion in peristriate cortex, precuneus, and posterior insula as compared to baboon-related expansion. Using the Davi130 parcellation (*8*), both cercopithecoid monkeys to chimpanzee expansion maps showed no relationship (baboon to chimpanzee: *r* = 0.11; *P* = 0.12 and macaque to chimpanzee: *r* = 0.08; *P* = 0.21) with age-related GM decline in chimpanzees (fig. S3B).

## DISCUSSION

We developed a data-driven framework for interspecies comparison and found a human-specific positive relationship between agerelated GM decline and expansion in the human brain compared to chimpanzees. In chimpanzees, on the other hand, there was no such brain age association for cerebral expansion relative to baboons and even a negative correlation with the brain’s expansion from macaque to chimpanzee. This suggests that the extensive expansion of the PFC and other cortical association areas in recent human evolution since splitting from a common ancestor with chimpanzees comes at the price of increased age-related vulnerability.

Unsupervised clustering of GM structure separately in humans and chimpanzees using OPNMF yields low-dimensional spatial parcellations matching coarse macroanatomy that provides a basis for cross-species comparison of brain organization. Our approach establishes brain parcellations based on species-specific information while identifying comparable organizational features between species. Both human and chimpanzee factorization solutions show overall hemispheric symmetry and spatial contiguity and align with known macroanatomical structures (*8*) as reported in previous applications of OPNMF in humans (*29*, *32*–*34*, *45*). The 17-cluster solution was selected on the basis of cross-species similarity and within-species reconstruction accuracy matching not only the granularity previously selected using OPNMF in human infants (*34*, *45*) but also resembling overall spatial structure of those OPNMF solutions. Similarities include parcels representing precuneus, insula, and superior parietal lobe, in the human factorization, while both the chimpanzee and human solutions show similarities to previous findings in the PFC and temporal pole (*29*, *32*, *34*, *45*). In addition, the superior parietal lobe and PFC parcels show similarities to spatial clusters that were reported to represent genetic influence on cortical thickness (*46*).

Independently in chimpanzees and humans, the OPNMF 17-cluster solution established a parcel representing ventrolateral, ventromedial, and orbital parts of the PFC, which shows the highest cross-species similarity as well greatest expansion in humans relative to chimpanzees. In previous studies, the PFC has been reported to be proportionally larger in humans compared to chimpanzees (*11*) while showing non-allometric scaling across anthropoid primates (*47*). With regard to macroanatomic pattern, this cluster of high expansion in humans includes regions of greater sulcal modification over primate evolution like the ventrolateral PFC, while such structural features remain comparable in the ventromedial PFC (*15*). The comparably coarse level of multivariate data-driven analysis presented here reveals similarity in the GM organization of these ventral and orbital subregions of the PFC. Furthermore, this region showed an exceptionally large age-related GM decline in humans alongside the high degree of expansion. This suggests that the greater expansion of PFC, which has been instrumental in evolutionary development in primate cognition (*48*), comes with the detriment of severe age-related GM decrease in humans, where the PFC plays an important role in higher-order cognitive functions, such as executive control (*25*, *49*), working memory (*50*), and language (*51*). The much greater ventral PFC expansion and age-mediated GM decline in humans compared to chimpanzees may be interpreted as additional dimension of the last-in-first-out hypothesis (*27*) in the developmental maturation to aging trajectory.

The relationship between human GM volume decline and cortical expansion indicates a link between the evolutionary development of specific cortical areas in humans and increased vulnerability to neurodegenerative processes. Such a relationship was not present in the expanded cortical regions of chimpanzee relative to baboons and macaques, although a significant GM decline was also present in chimpanzees (*8*). The main difference between humans and chimpanzees seems to be the more prominent expansion in sensorimotor regions in chimpanzees relative to the cercopithecoid monkeys, whereas regions of human cortical expansion relative to chimpanzees is generally observed in more multimodal association regions. This may be related to chimpanzees’ improved abilities for tool use as compared to cercopithecoid monkeys (*52*). Human multimodal cortical areas are characterized by lower neuronal cell density, as well as higher dendritic branching and spine numbers of pyramidal neurons (*53*, *54*). Compared to other great apes, the human brain has a large neuropil fraction in the frontal pole (*55*) and the anterior insula (*56*). The neuropil fraction represents the space surrounding cell bodies occupied by dendritic and axonal interconnectedness. Both these areas (frontal pole and insula) show a combination of large expansion and age-related changes on GM volume (Fig. 4A) in humans. With dendritic reduction and synapse loss being characteristics of normal aging processes (*57*), the relatively increased neuropil space of human association cortex may partly explain the aging-expansion relationship we observe. The medial and orbito-frontal cortex and the insula that displayed large expansion and age-mediated GM decline have been previously found to have high deterioration of glucose metabolism and large accumulation uptake of β amyloid in human aging (*58*–*60*).

The large expansion in frontal and parietal regions is comparable to studies using cortical surface measures to estimate the expansion from chimpanzee to human (*24*) as well as macaque to human (*28*). In addition, using a latent space from functional MRI data, Xu and colleagues (*61*) have also shown high expansion in parietal and frontal areas. In their analysis, the chimpanzee to human surface expansion map showed less expansion in the ventral medial PFC and the insula compared to our results. The difference could relate to our utilization of volumetric maps that were initially created voxel-wise and then masked with our comparative 17-cluster solution, while Wei and colleagues (*24*) used a higher granularity human surface atlas and mapped the expansion value within this atlas space. The macroanatomical surface and function expansion maps previously reported between macaque and human both show greater expansion in the lateral temporal lobe and less in the ventral medial PFC compared to our chimpanzee to human map. This may again relate to the different brain data type for the lateral temporal lobe, we showed greater expansion in the cercopithecoid monkey to chimpanzees, and this may show that the expansion of the lateral temporal lobe relates to monkey to great ape expansion and not specifically to humans.

Several limitations need to be considered when interpreting the results presented in this study. First, our voxel-based morphometry (VBM) analysis was limited to structural information present in the T1w contrast images and our method’s accuracy of tissue segmentation. It does not include a T2w contrast or additional modalities like structural connectivity or functional dynamics. Such complimentary information provided by additional modalities will help to establish a more comprehensive basis for cross-species comparison. GM changes can additionally be measured through surface-based modeling of cortical thickness and surface area that generally show a very similar decline with age as volumetric analyses (*62*). Furthermore, VBM-derived volume estimates are vulnerable to partial volume effects at the border between tissues and can lead to an underestimation of GM volume, particularly in regions of increased myelination like motor and occipital cortices. These issues can be minimized by accurate tissue segmentation assisted through representative a priori templates as were used in this study with the JunaChimp templates (*8*) alongside the well-tested CAT12 human processing pipeline. Another limitation of the current study to note is that the chimpanzee MRI sample features a marked sex bias with 67% of the subjects being female; this sex imbalance increasing to 86% over 40 years of age. Unfortunately, this bias largely prevents the separate modeling of sex effect in brain aging. All inferences of age-related GM atrophy are driven by cross-sectional data in both the chimpanzee and the matched human samples with moderate age distributions. A longitudinal study design can provide additional information on specific trajectories of structural brain changes throughout the life span. To infer the unique relationship in human evolution between aging and regions of cortical expansion, we were limited by the availability of representative templates of one great ape species, the chimpanzee and two cercopithecoid monkey species, the macaque, and the baboon. Further research including using additional primate species in a broader phylogenetic investigation will establish a better understanding at which evolutionary state these aspects of the neurobiology of aging occur.

In conclusion, we used a data-driven comparative analysis framework that reveals structural features of great ape brain organization to study human brain aging in a comparative context. The species-specific GM parcellations using OPNMF contain both inter-and intraspecies anatomical features and provide the basis for macroanatomical cross-species comparison. Using the optimal 17-cluster solution, we found a significant relationship between evolutionary brain expansion and brain aging in humans only. Regions with high cerebral expansion in humans relative to chimpanzees showed extensive GM decline. A weaker but similar pattern of brain aging in chimpanzees did not show this association with brain expansion relative to baboons nor to macaques. These findings suggest that recent evolutionary changes in human brain organization involving differential expansion of multimodal association regions may increase these regions’ unique vulnerability to age-related neurodegenerative processes, in particular the ventral PFC.

## MATERIALS AND METHODS

### Sample description

The chimpanzee T1w MRI scans were provided by the NCBR containing brain scans of 223 captive animals (137 females; 9 to 54 years old; mean age = 26.9 ± 10.2 years). The chimpanzees were housed at the National Center for Chimpanzee Care (NCC) at The University of Texas MD Cancer Center (*N* = 147; 1.5 Tesla G.E echo-speed Horizon LX scanner) or the Emory National Primate Research Center (ENPRC; *N* = 76; 3.0 Tesla Siemens Trio scanner). The MRI scanning procedures for chimpanzees at both the NCC and ENPRC were designed to minimize stress for the animals. Data were acquired with ethics approval (#YER-2001206) and were obtained before the 2015 implementation of the US Fish and Wildlife Service and National Institutes of Health regulations governing research with chimpanzees. Image quality control (QC) was conducted by assessing outliers in GM intensity values over the whole brain after CAT12 template normalization. One hundred ninety-four chimpanzees (130 females; 9 to 54 years old; mean age = 26.2 ± 9.9) passed QC. To minimize the effect of old age on the OPNMF clustering solutions, subjects over 50 were removed for a final sample of 189 chimpanzees (126 females; 9 to 50 years old; mean age = 25.6 ± 9.1).

The human structural T1w MRI scans were provided by the IXI dataset. This publicly available dataset was chosen for optimal comparability with the NCBR chimpanzee MRI scans, as it also contains images from two sites with both 1.5 and 3 Tesla magnets, respectively. IXI also has a comparable distribution of age and sex and consists of 565 healthy subjects (314 females; 20 to 86 years old; mean age = 48.69 ± 16.46 years) without missing metadata. To further match the two NCBR scanners, only subjects from the Hammersmith Hospital (*N* = 181; 3.0 Tesla Philips Medical Systems Intera scanner) and Guy’s Hospital (*N* = 315; 1.5 Tesla Philips Medical Systems Gyroscan Intera scanner) were considered. All 496 subjects (270 females; 20 to 86 years old; mean age = 49.57 ± 16.28 years) passed QC after CAT12 processing. For further comparability with the chimpanzee sample, very old IXI subjects over 75 years of age were omitted for the construction of the OPNMF solutions resulting in the final sample of 480 subjects (262 females; 20 to 74 years old; mean age = 48.7 ± 16.5). We used the eNKI open neuroimaging dataset (*44*) for replication of the main result of aging-expansion relationship in an independent life-span sample. The eNKI scans were acquired using a single 3T scanner (Siemens Magnetom TrioTim), and T1w images were obtained using a magnetization-prepared rapid acquisition gradient echo sequence with repetition time = 1900 ms and 1-mm isotropic voxels. Following QC, 52 subjects from eNKI (*44*) were removed due to poor CAT12 QC ratings, and 765 subjects (502 females; 6 to 85 years old; mean age = 39.8 ± 22.2) remained for our replication analysis. The NCBR chimpanzee and IXI and eNKI datasets are cross sectional in nature.

### Image processing

The chimpanzee (NCBR) and human (IXI and eNKI) samples were preprocessed using CAT12 (*36*) (Computational Anatomy Toolbox; www.neuro.uni-jena.de/cat/; r1725) in SPM12 (Statistical Parametric Mapping; www.fil.ion.ucl.ac.uk/spm/software/spm12/; v7487). The NCBR sample was processed using a recently established chimpanzee-specific processing pipeline (*8*), while IXI and eNKI samples used the default human processing pipeline with high-accuracy shooting registration (*63*). The general steps of preprocessing were as follows: First, the single subject images are linearly registered to the template space using 12 df. Then, each image is initially segmented into the three tissue types, GM, white matter, and cerebrospinal fluid, using a species-specific tissue probability map. Next, the resulting three tissue maps are nonlinearly registered to the population template in five steps using templates with increasing sharpness (decreasing smoothing) (*63*). Last, the resulting deformation fields are used to modulate GM probability maps to conserve the original local volume of each brain in template space. Following preprocessing, the modulated GM maps for each species were down-sampled (2- and 3-mm resolution) and smoothed (4- and 6-mm full width half maximum) in the NCBR and human samples, respectively. Last, GM masks at 0.3 probability for chimpanzees and 0.2 for human samples were applied encompassing the cortex and basal ganglia for further processing.

QC within each sample was conducted by computing the sample’s inhomogeneity of GM with CAT12. The modulated GM maps with a mean correlation below 2 SDs were flagged for visual inspection. The flagged images were then removed if they contained tissue misclassification, artifacts, irregular deformations, or very high intensity values. This process was repeated a second time with the passed images in the chimpanzee sample only, and no images were removed in the IXI sample. Following the second iteration in the chimpanzee sample, no more images were flagged as outliers. Following QC, 194 (130 females, 9 to 54 years old; mean age = 26.2 ± 9.9) chimpanzees and 496 (270 females, 20 to 86 years old; mean age = 49.57 ± 16.28 years) human T1w images qualified for further investigation.

To ascertain overall accuracy of the cross-species, chimpanzee to human deformation maps, visual quality assurance was conducted. For this purpose, the macroanatomical chimpanzee parcellation, Davi130 (*8*), was deformed to the human Montreal Neurological Institute (MNI) space and visually inspected for large systematic misalignment of comparable macroanatomical landmarks, independently by two authors S.V. and R.D. (fig. S1). There are some slight misalignment of gyri, and the superior cerebellum and posterior parts of the superior frontal gyrus have moved slightly too far superiorly. As the OPNMF parcels used here are very large and as additional GM masking was conducted, these small differences did not relevantly affect our analysis. On the basis of this assessment, we deem the cross-species deformation map appropriate to project chimpanzee-derived macroanatomical features onto a human template brain. In addition, we visually inspected the deformed chimpanzee OPNMF cluster solutions in MNI space for visible artifacts.

### OPNMF and granularity selection

For cross-species comparison, we created a whole brain parcellation for each great ape species by using an orthogonal modification of non-negative matrix factorization (NMF), the OPNMF (*29*, *30*). This kind of factorization approach requires to a priori set a number of factors to be estimated, resulting in this number of clusters. NMF factorizes a data matrix (*X*) with dimensions *m* by *n* (GM voxels by subjects) into a factor matrix *W* (*m* by *k*, voxels by factors) and a subject-specific factor weight matrix *H* (*k* by *n*, factors by subjects) with non-negative elements in all three matrices. The construction of the factor (*W*) and weight (*H*) matrices is achieved by minimizing the reconstruction error, which is given by the difference between multiplication of the factorized matrices (*W* and *H*) and the original input matrix. Thereby, NMF separates units of covariance in the input data and creates a part-based representation of the original input data through the resulting factors (*64*). Technically, OPNMF factorizes the data matrix by solving the minimization problem

$$\|X-{\mathrm{WW}}^{T}X\|\;\text{subject to}\;W^{T}W=I;W\geq 0$$ (1)

where ||.|| refers to the squared Frobenius norm and *I* denotes the identity matrix. To first initialize the *W* matrix for the factorization, we used non-negative double singular value decomposition (*65*) which encourages sparsity of factors. Subsequently, *W* is iteratively updated (*k* = 10,000) until it reaches an optimal solution (*30*) with the multiplicative update rule

$${W\prime }_{\mathrm{ij}}=W_{\mathrm{ij}}\frac{{\left({\mathrm{XX}}^{T}W\right)}_{\mathrm{ij}}}{{\left({\mathrm{WW}}^{T}{\mathrm{XX}}^{T}W\right)}_{\mathrm{ij}}}$$ (2)

The final step is to project *X* onto *W* to calculate the weight matrix *H* as follows

$$H=W^{T}X$$ (3)

OPNMF was chosen instead of the original NMF as it provides several advantages when representing structural T1w MRI data as a small number of structural covariance factors (*29*, *34*, *35*). OPNMF is different from standard NMF on how it constructs the factor loading weights of the *H* matrix. In OPNMF, it is estimated by projecting the input matrix (*X*) onto the factor matrix (*W*), while in standard NMF, the weight matrix (*H*) is estimated separately. Therefore, in OPNMF, all factors participate in the reconstruction of all data points, while in NMF, a subset of factors is involved in reconstructing a subset of data points leading to greatly less overlap of factors and more sparsity in OPNMF. This leads to the creation of factors that are spatially continuous with minimal overlap, here providing a low-dimensional representation of the underlying GM. Each voxel is assigned a specific cluster in the brain by using a winner takes all approach when projecting the factors back onto the brain to create the final solution. As the number of factors is assigned a priori for OPNMF, a data-driven approach was used to determine the number of clusters accounting for accuracy and stability of solutions within species and spatial similarity between species. As the reconstruction error quantifies how well the factorization solution represents, it allows us to determine the improvement of an OPNMF solution when increasing the number of factors. A plateau in the improvement of the reconstruction error with additional factors likely shows the modeling of noise rather than signal. Accordingly, we averaged the change in reconstruction error over 100 bootstrapped implementations of OPNMF across a range of factor numbers (2 to 40 steps of 1) to provide a stable indication of the reconstruction accuracy change for each species separately. For interspecies comparability, we selected the factor solution with the highest spatial similarity between species using the ARI after deformation of the chimpanzee OPNMF solutions to the human MNI space.

### Template processing and expansion maps

With CAT12, we establish cross-species deformation maps, by using population-based templates of one species and the processing pipeline of another target species resulting in the approximation of relative cross-species brain expansion. The use of representative average brain templates for each species aids generalizability of the derived expansion maps. In addition, their high tissue contrasts enable accurate segmentation and registration. We used well-tested, species-specific processing pipelines for chimpanzees (*8*) and humans to create expansion maps for both. The chimpanzee to human expansion map was created using the chimpanzee template in the human processing pipeline. Baboon and macaque to chimpanzee expansion maps were created using the chimpanzee pipeline (*8*) with representative templates of baboon (*38*) and macaque (*39*–*41*) monkeys. For the latter, we averaged the expansion maps of three commonly used macaque templates (*39*–*41*) to encompass intersample variation. Quality assessment was performed on all deformation and expansion maps by assessing overall smoothness and location of representative macroanatomical structures.

### GM aging and expansion analyses

Age-related GM decline in humans and chimpanzees was analyzed using cross-sectional data. The relationship between aging and relative regional expansion in chimpanzees and humans was assessed using the comparable OPNMF clustering solution. Parcel-wise linear regression models were computed for both species to estimate agerelated GM atrophy. The age range of the IXI sample was matched to the chimpanzee sample to improve comparability by accounting for the assumed difference in aging processes (human ≈ 1.15× chimpanzee) (*42*). This represented the chimpanzee maximum age-matched sample that included 304 human subjects (females = 150; 20 to 58 years old; age = 39.0 ± 11.0). A 1:1 human-chimpanzee matched sample was based on age (adjusted), sex, and scanner field strength to ensure the robustness of our findings with respect to the age and sex sample differences between species. The matching was conducted using the MatchIt R package (*66*) by implementing the “optimal” algorithm (*67*), which determines the optimal matched dataset that has a sum of pairwise distance as small as possible. This established a matched human sample with 189 subjects (females 122; age range, 20 to 61 years old; mean age = 33.2 ± 8.7). The chimpanzee sample contains 189 subjects (126 females; 9 to 50 years old; age = 25.6 ± 9.1). Average GM volumes for each OPNMF parcel from the chimpanzees and humans were entered into a regression model for each species as the independent variable with age, sex, TIV, and scanner field strength as the dependent variables. Whole brain GM was represented as a percentage of TIV to account for differences in brain size in the whole brain GM age regression models in both species. Significant age-mediated GM decline was determined at *P* ≤ 0.05 and for parcel-wise analyses, following correction for multiple comparisons using family-wise error (FWE) (*43*). The parcel-wise age model *t*-statistics in the human sample were compared with the chimpanzee to human expansion, while chimpanzee age-mediated GM decline was compared with the baboon and macaque cross-species expansion. Cross-species expansion was estimated by computing the mean expansion of each parcel for the various cross-species expansion maps and *z*-scored to represent the interregional interspecies expansion. Significance of Pearson’s correlation and difference between correlations were determined through permutation testing (*k* = 100,000) at *P* ≤ 0.05. Last, the parcel-wise age-related GM decline in humans was also analyzed in the independent eNKI (*44*) life-span dataset (*n* = 765, 502 females; 6 to 85 years old; mean age = 39.8 ± 22.2) and correlated with the chimpanzee to human expansion to replicate the main finding.
