# Supplementary material for: The uniqueness of human vulnerability to brain aging in great ape evolution
Source: Sci Adv. 2024 Aug 28;10(35):eado2733. doi: 10.1126/sciadv.ado2733 (PMC11352902; doi:10.1126/sciadv.ado2733)
Supplement: Supplementary file 1 — Figs. S1 to S4 [file sciadv.ado2733_sm.pdf]

Science Advances
AAAS

Supplementary Materials for

The uniqueness of human vulnerability to brain aging in great ape evolution

Sam Vickery *et al.*

Corresponding author: Sam Vickery, s.vickery18@gmail.com; Felix Hoffstaedter, f.hoffstaedter@fz-juelich.de

Sci. Adv. **10**, eado2733 (2024)
DOI: 10.1126/sciadv.ado2733

This PDF file includes:

Figs. S1 to S4

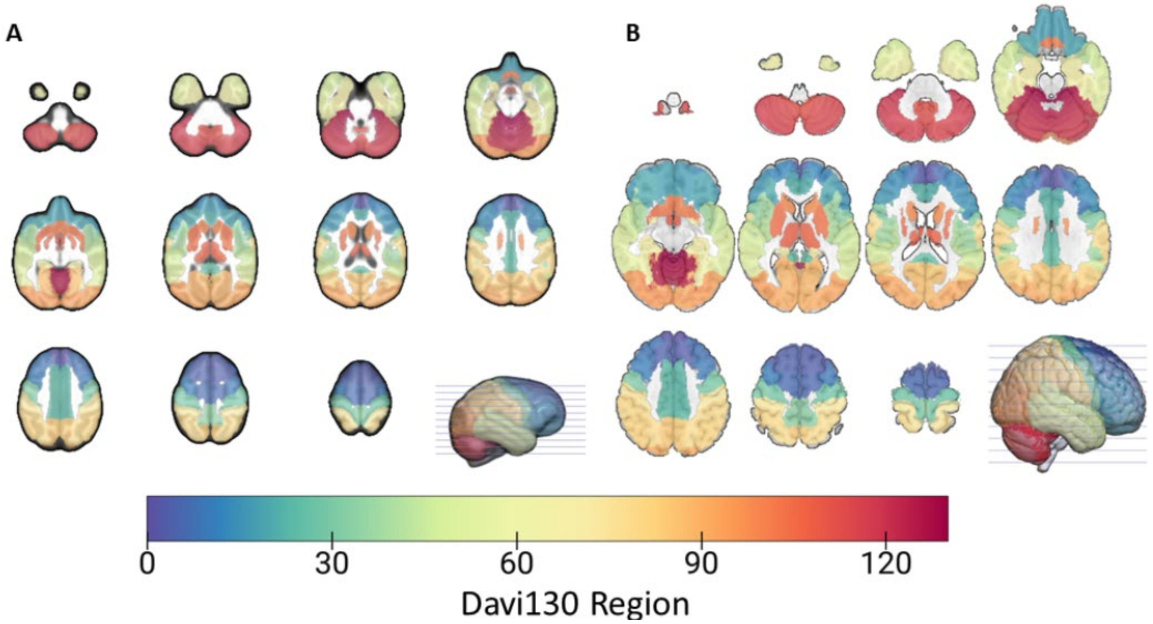

Figure S1. Deformation map quality control. Davi130 chimpanzee parcellation in chimpanzee (A) and human (B) template space used for visual quality control of chimpanzee to human deformation map.

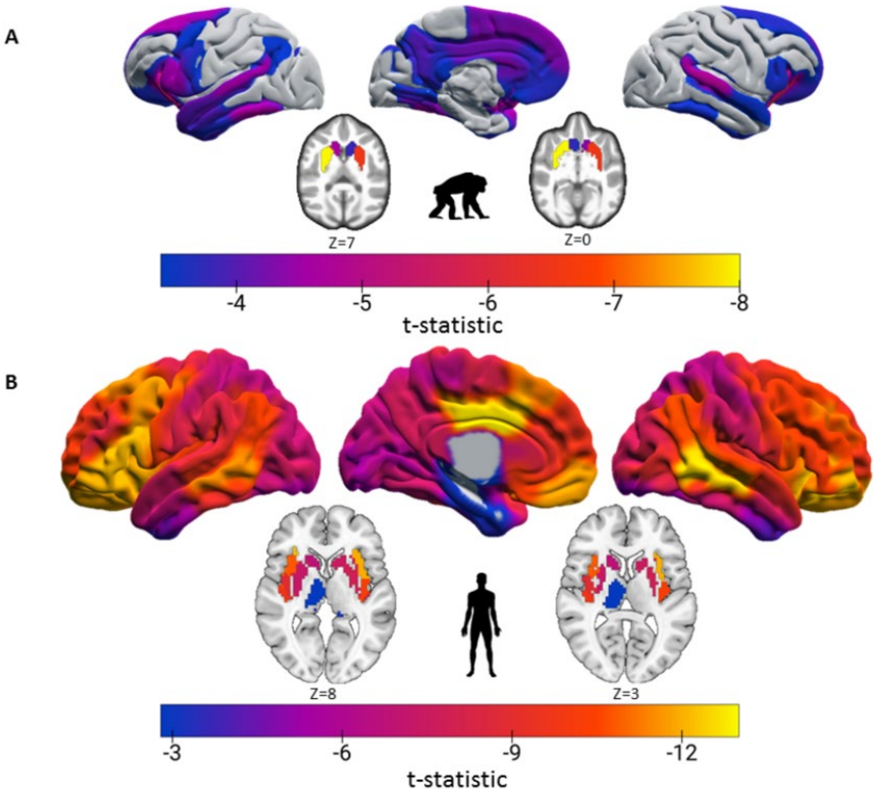

Figure S2. Comparison of aging effect on GM volume. Aging effect on GM volume across all cortical and sub-cortical Davi130 regions in chimpanzees (A) and human (B) samples. Significant regions at $p \leq 0.05$ are presented following correcting for multiple comparisons using FWE.

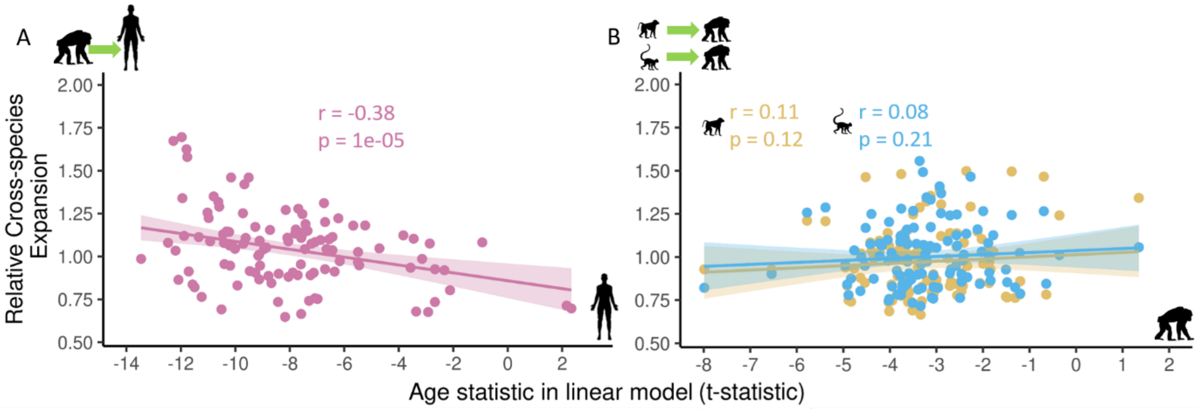

Figure S3. Aging – expansion comparison (Davi130). Scatter plots showing of cross-species expansion and aging effect between A – chimpanzee to human expansion and human aging effect (Pink), B – macaque to chimpanzee expansion and chimpanzee aging effect (Blue) and baboon to chimpanzee expansion and chimpanzee aging effect (Yellow). Significance (p) of correlation (Person's r) for cross-species expansion and aging effect relationship is determined by permutation testing (k = 100 000). Note, two Davi130 regions presented positive t-statistics and therefore could not be inversed as seen in all other age regression models.

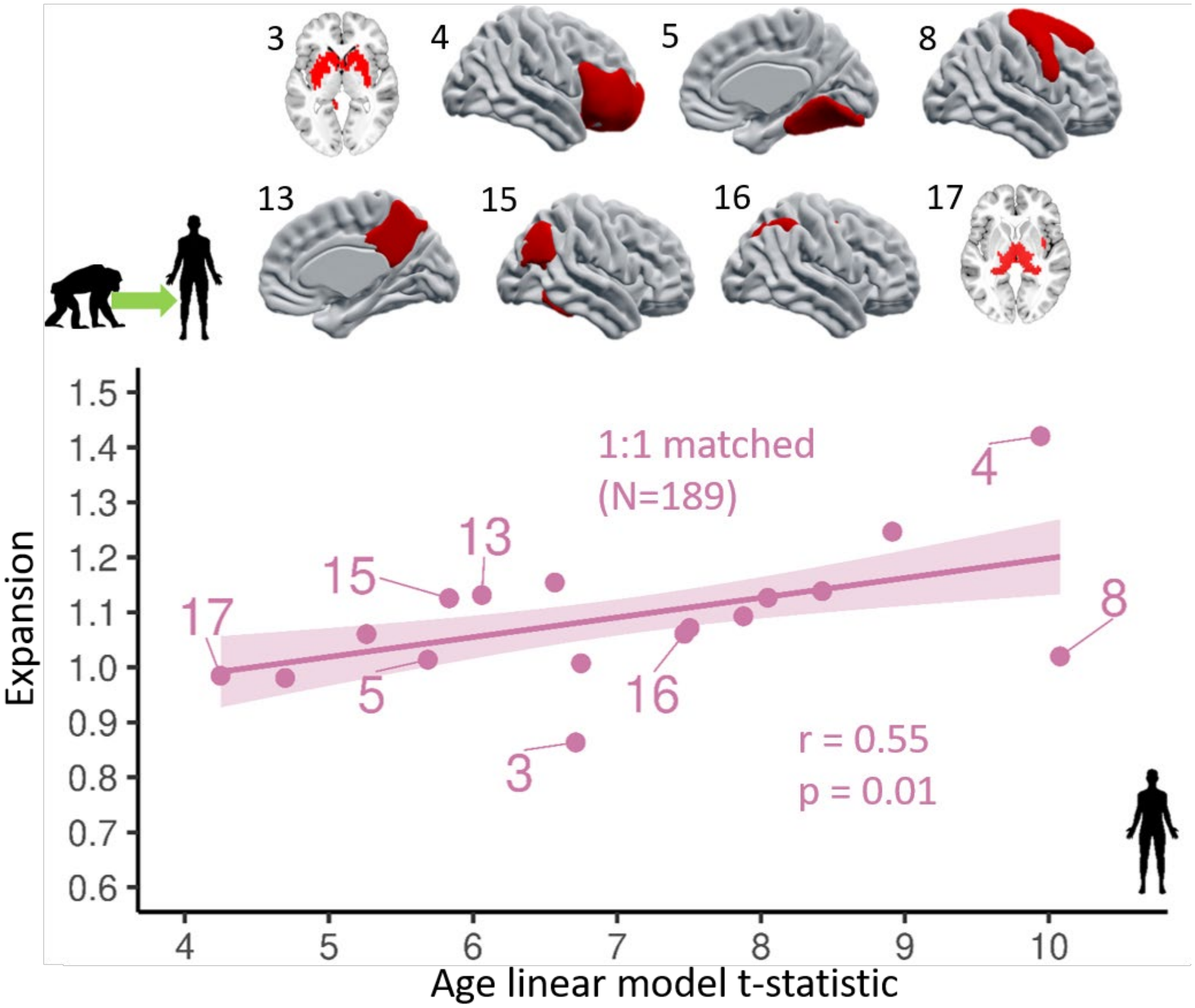

Figure S4. Aging – expansion comparison (1:1 matched). Scatter plots showing of cross-species expansion and aging effect using the whole IXI sample OPNMF 17-factor solution to extract aging effect for each factor using the 1:1 matched IXI sample (n=189, 112 females; 20 – 61 y/o; mean age = 33.2 ± 8.7). A selection of OPNMF factors are projected onto volume slice or rendering of the MNI human template. Significance (p) of correlation (Person's r) for cross-species expansion and aging effect relationship is determined by permutation testing (k = 100 000).
